# Complications métaboliques après transplantation rénale à partir du donneur vivant: expérience du CHU Ibn Sina de Rabat

**DOI:** 10.11604/pamj.2014.18.166.1213

**Published:** 2014-06-19

**Authors:** Hala Mouram, Loubna Benamar, Naima Ouzeddoune, Rabia Bayahia, Fatima Ezaitouni

**Affiliations:** 1 Service de néphrologie, Dialyse, Transplantation rénale, CHU Ibn Sina, Rabat, Maroc

**Keywords:** Transplantation rénale, diabète, dyslipidémie, obésité, hyperuricémie, risque cardiovasculaire, prévention, traitement, retour en dialyse, décès, kidney transplant, diabetes, dyslipidemia, obesity, hyperuricemia, cardiovascular risk, prevention, treatment, return to dialysis, death

## Abstract

La transplantation rénale (TR) constitue le traitement de choix de l'insuffisance rénale chronique terminale. Les complications métaboliques après TR (diabète, dyslipidémie, hyperuricémie, obésité), en grande partie liées au traitement immunosuppresseur, deviennent une préoccupation car elles constituent un facteur de risque de morbimortalité et de perte fonctionnelle du greffon. Le but de notre étude est d’évaluer la fréquence de ces anomalies après TR. Il s'agit d'une étude rétrospective incluant tous les patients ayant bénéficié d'une première TR par donneur vivant (DV) de Juin 1998 à Décembre 2010. Nous avons recensé les données démographiques propres au receveur, le traitement immunosuppresseur après TR. Les paramètres clinico-biologiques recueillis sont (index de masse corporel (IMC), glycémie à jeun, hémoglobine glyquée, CT, C-HDL, C-LDL, TG, acide urique). Soixante dix patients ont été colligés, l’âge moyen est de 36.3 + /-9.6 ans (21 à 62) avec un sex ratio de 0.5. Quinze patients (21.4%) étaient hypertendus avant la TR et 2.9% avaient une néphropathie diabétiques. L’âge moyen du donneur est de 47.5 + /-10.2 ans (20-65). Le traitement immunosuppresseur pendant la phase d'induction était une trithérapie associant corticostéroïdes, anticalcineurines chez tous les patients et mycophénolate mofétil chez 68.6% et azathioprine dans 31.4% des cas. L'IMC moyen était de 24.1 + /-4.0 (16.9 à 37), 33% des patients étaient considérés en surpoids dont 21.8% en obésité. L'hypercholestérolémie, a été retrouvée chez 25 patients soit 36%. Presque la moitié des patients (48.5%) avaient une hyperuricémie. Quatre patients ont développé un diabète après TR soit 6% des cas. La perte du greffon a été notée chez 12 patients et 2 patients sont décédés dont un avec un greffon fonctionnel. En analyse univariée, l'hyperuricémie et la dyslipidémie ont été considérées comme facteur de risque de perte du greffon et retour en dialyse avec p = 0.024 et 0.021 respectivement. Les complications métaboliques après TR sont fréquentes et méritent une attention particulière car elles représentent un facteur de morbi-mortalité. L’éducation précoce du patient greffé est nécessaire et s'appuie sur une prise en charge multidisciplinaire impliquant les néphrologues, diététiciennes, psychologues et médecins généralistes.

## Introduction

Les complications métaboliques après la transplantation rénale (TR) sont fréquentes et méritent une attention particulière car elles représentent des facteurs de morbi-mortalité cardiovasculaire. L'installation de ces désordres est précoce et leurs conséquences à long terme peuvent être vitales [1]. L'objectif de notre étude est d’évaluer les complications métaboliques après TR, leur fréquence, les facteurs ayant influencé leur survenue ainsi que leur prise en charge, leur évolution et le retentissement à court et à long terme sur la fonction du greffon.

## Méthodes

Il s'agit d'une étude rétrospective incluant tous les patients ayant bénéficié d'une première TR par donneur vivant (DV) de Juin 1998 à Décembre 2010 à l'unité de TR au CHU Ibn Sina de Rabat. Nous avons recensé les caractéristiques propres au receveur (l’âge, sexe, antécédents personnels et familiaux, néphropathie initiale, modalités de dialyse), au donneur (âge, sexe, antécédents), le traitement immunosuppresseur après TR (phase initiale et d'entretien). Les paramètres clinico-biologiques recueillis étaient (index de masse corporel (IMC), glycémie à jeun, hémoglobine glyquée, CT, C-HDL, C-LDL, TG, acide urique). Nous avons définit un surpoids et une obésité par un IMC supérieur à 25 et 30kg/m^2^ respectivement. Le diabète a été définit par une glycémie à jeun supérieur à 1.26 g/l, une dyslipidémie par un taux de cholestérol total C-LDL supérieur à 1,3 g/l et une hyper uricémie par un taux d'acide urique supérieur à 65 g/l chez la femme et 80 g/l chez l'homme. Nos définitions sont validées par l'OMS et l'AFSSAPS [2]. L'analyse statistique est réalisée à l'aide du logiciel SPSS 10. Les variables quantitatives sont exprimées en moyennes – écart types quand elles répondent à la loi normale et en médianes - interquartiles: 25-75 quand elles sont hors la loi normale. Les variables qualitatives sont exprimées en pourcentage. La comparaison des moyennes quantitatives est réalisée à l'aide du test t de Student et le test exact de Fisher et la comparaison des variables qualitatives à l'aide du test de Chi-carré.

## Résultats

Sur une période de 12 ans, nous avons colligé 70 patients ayant bénéficié d'une première TR par DVA dans l'unité de TR du CHU Ibn Sina de Rabat. L’âge moyen de nos patients est de 36.3 ±9,6 ans (21-62), avec un sex ratio de 0.52 (34.3%% des hommes et 65,7% des femmes). Quinze patients (21%) étaient hypertendus avant la TR sous traitement médical. La néphropathie initiale est une glomérulonéphrite chronique dans 20.6% des patients et 2.9% des patients avaient une néphropathie indéterminée, cependant, elle reste indéterminée chez 48.6% des patients. L'hémodialyse conventionnelle intermittente a été la technique d’épuration extrarénale chez la majorité de nos patients (97.1%), deux patients étaient en dialyse péritonéale. La durée médiane de dialyse était de 30,6 mois (2 à 120).

Concernant le donneur, l’âge moyen est de 41,5±10.2 ans (20 à 65ans). Le greffon est implanté en fosse iliaque gauche chez 63 patients (90%). Les complications métaboliques observées sont: un diabète de novo, dyslipidémie, hyperuricémie et une obésité dans respectivement 5.8%,36%, 48.5% et 33% des cas

**Diabète:** Quatre patients (5.8%) ont développé un diabète après un délai médian de 15 mois (2-36mois) de la TR. Trois patients ont nécessité le recours à l'insulinothérapie et un patient a été traité par des antidiabétiques oraux. Le diabète est bien équilibré chez tous les patients et aucune complication micro ou macroangiopathiques n'a été observée. Deux patients ont présenté un épisode de rejet aigu cellulaire avec une bonne évolution après réadaptation du traitement immunosuppresseur. Aucun retour en dialyse n'a été noté. Dans notre étude, la survenue du diabète était indépendante de l’âge du receveur, de la dose de corticoïdes, de l'infection par le VHC et l'utilisation de la tacrolémie.

**Dyslipidémie:** La dyslipidémie a été retrouvée chez 25 des patients après un délai médian de 24 mois (6à180) de la TR. Le taux moyen de TG est de 1.9 ±-0.7 g/l avec un cholesterol-LDL moyen de 1.6±0.2g/l. Parmi ces patients, neuf sont en surpoids et 3 en obésité. L’évolution a été marquée par une perte du greffon rénal et retour en dialyse chez 8 patients. Deux patients sont décédés dont un avec un greffon fonctionnel.

**Obésité:** Dans notre population, l'IMC moyen est de 24.1±4 kg/m, 23(32.8%) patients sont en surpoids (IMC > 25 kg/m^2^) dont 5 en obésité morbide (IMC > 30Kg/m^2^). La dyslipidémie est retrouvée chez 10 patients (43.5%) et 83% de ces malades sont hypertendus.

**Hyperuricémie:** Elle est retrouvée chez 34 patients soit 49%. Aucun patient n'a présenté une symptomatologie clinique en rapport avec cette complication. Une perte du greffon a été observée chez 2 patients.

**Facteurs pronostiques:** La dyslipidémie et l'hyperuricémie représentent des facteurs de risques associés à une perte du greffon et retour en dialyse dans notre étude (p = 0.024 et 0.021 respectivement).

## Discussion

Le transplanté doit être considéré comme un sujet à haut risque cardiovasculaire. Tous les moyens doivent être mis en aeuvre pour améliorer l'hygiène de vie, lutter contre l'obésité, dépister et traiter un diabète sucré ou une hyperlipidémie [1].

En effet, La dyslipidémie après TR est fréquente. Son incidence varie entre 30 et 42% selon les séries [3, 4, 5]. Il s'agit le plus souvent d'une dyslipidémie mixte, associant hypercholestérolémie et hypertriglycéridémie, elle-même favorisée par les troubles du métabolisme glucidique. Les traitements immunosuppresseurs jouent un rôle important dans la survenue de dyslipidémie en particulier les corticoïdes qui provoquent une insulinorésistance périphérique. Le traitement repose dans un premier lieu sur les mesures hygiéno-diététiques puis par l'utilisation prudente des statines surtout avec la ciclosporine. La pravastatine et la simvastatine sont les deux statines les plus indiquées chez les transplantés car elles n'inhibent pas le CYP3A4 et ont moins de toxicité musculaire [4]. Les transplantés dont le taux de LDL-C est supérieur à 3 mmol/l doivent bénéficier d'un traitement hypolipémiant, surtout lorsqu'ils ont d'autres facteurs de risque ou des antécédents cardiovasculaires [3, 4]. Dans notre série, la dyslipidémie est considérée comme un facteur de risque majeur de retour en dialyse. Le diabète après TR est une complication fréquente et grave. Selon les données de la littérature, son incidence est de 13% [5, 6, 7, 8]. L'existence d'un effet dose-dépendant est bien établie pour les corticoïdes et le tacrolimus qui est plus diabétogène que la ciclosporine [6, 7]. Le traitement s'apparente à celui du diabète de type 2: règles hygiéno-diététiques, antidiabétiques oraux si la fonction rénale le permet et insulinothérapie [7, 8]. Dans notre étude aucun facteur de risque associé n'est retrouvé en analyse statistique.

L'hyperuricémie est très fréquente après TR. Son incidence varie de 18 à 85% selon les études [9]. La population des transplantés cumulent plusieurs facteurs de risque, en particulier l′hypertension, l′insuffisance rénale et la prise de diurétiques, les études comparatives ont démontré que la ciclosporine constitue le facteur de risque principal d′hyperuricémie et par conséquent de goutte dans cette population. Le traitement inclut les règles hygiéno-diététiques et surtout un traitement hypo-uricémiant. L'allopurinol peut être utilisé en adaptant la posologie à la fonction rénale [9, 10]. Il reste formellement contre-indiqué en association avec l'azathioprine car il inhibe la xanthine oxydase nécessaire au catabolisme de l'azathioprine et peut être responsable d'une pancytopénie. Dans notre étude, l'hyperuricémie représente un facteur de risque de perte du greffon statistiquement significatif.

En ce qui concerne la surcharge pondérale après transplantation rénale, plusieurs facteurs prédictifs sont proposés: un IMC élevé, tant chez le donneur que chez le receveur avant la TR, rejet aigu et de fortes doses cumulées de corticostéroïdes. Il n'est actuellement pas encore établit de relation entre l'excès pondéral et la survie à long terme du greffon [11]. Néanmoins, sa propension à révéler, ou aggraver, une HTA, un diabète et/ou une dyslipidémie, incite à le prendre en compte précocement, au même titre que ces autres complications. Dans notre population, une obésité morbide est retrouvée chez 7% des patients.

## Conclusion

Au total, les complications métaboliques après TR son fréquentes. Elles méritent une attention toute particulière de la part des équipes assurant le suivi de ces patients. L’éducation précoce du patient est une priorité. Elle doit s'appuyer sur une prise en charge multidisciplinaire impliquant diététiciennes, psychologues, médecins généralistes et association de greffés.
